# Cell-to-cell transmission promotes the emergence of double-drug resistance

**DOI:** 10.1093/ve/vead017

**Published:** 2023-03-11

**Authors:** Koichi Saeki, Akira Sasaki

**Affiliations:** Department of Computational Biology and Medical Sciences, Graduate School for Frontier Sciences, The University of Tokyo, Kashiwa, Chiba 277-0885, Japan; Research Center for Integrative Evolutionary Science, The Graduate University for Advanced Studies, SOKENDAI, Hayama, Kanagawa 240-0193, Japan; Evolution and Ecology Program, International Institute for Applied Systems Analysis, Laxenburg A-2361, Austria

**Keywords:** cell-to-cell transmission, intra-host viral dynamics, multiple-drug-resistance, Wright-Fisher model with killing, cell dynamics

## Abstract

The use of multiple antivirals in a single patient increases the risk of emergence of multidrug-resistant viruses, posing a public health challenge and limiting management options. Cell-to-cell viral transmission allows a pair of viruses that are each resistant to a single drug to persist for a prolonged period of passages although neither can survive alone under double-drug treatment. This pair should then persist until they accumulate a second mutation to generate resistance to both drugs. Accordingly, we here propose a hypothesis that viruses have a much higher probability of developing double-drug resistance when they are transmitted via a cell-to-cell mode than when they are transmitted via a cell-free mode through released virions. By using a stochastic model describing the changes in the frequencies of viral genotypes over successive infections, we analytically demonstrate that the emergence probability of double resistance is approximately the square of the number of viral genomes that establish infection times greater in cell-to-cell transmission than in cell-free transmission. Our study suggests the importance of inhibiting cell-to-cell transmission during multidrug treatment.

## Introduction

Simultaneous administration of multiple antiviral agents in a single patient is common. For example, during antiretroviral therapy for human immunodeficiency virus type 1 (HIV-1) infections, the standard first-line regimens combine multiple reverse transcriptase inhibitors or reverse transcriptase inhibitors with protease or integrase inhibitors. However, the use of multiple antivirals in a single patient increases the risk of the emergence of multidrug-resistant viruses, leading to treatment failure and limited treatment options, thereby contributing to a public health crisis. Therefore, there is a need to develop new antivirals or antiviral strategies that suppress the emergence of multidrug resistance.

**Figure 1.**
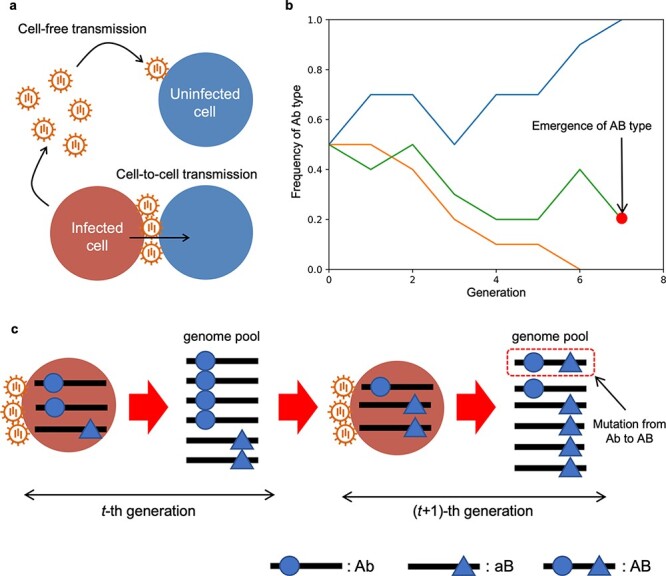
Overview of the model. (A) Cell-to-cell and cell-free viral transmission modes. We assume that the density of virions in a host body would be so low that at most one virion can infect a target cell. (B) Example trajectories of the frequency of Ab type simulated by the WF model with killing, where $n = 10$ and $u = {10^{ - 4}}$. In one case, an AB virus was chosen as one of the genomes for the next generation, and then, the process is killed. (c) The scheme of the changes in viral genome frequencies over successive infections. In this example, three viral genomes coinfect a target cell ($n = 3$). A genome pool of $t$-th generation is produced by replication in an infected cell, and the genomes for a newly infected cell ($\left( {t - 1} \right)$-th generation) are randomly sampled from the genome pool. A viral genome in the genome pool may gain a drug resistance by mutation with some probability, $u$.

Virus transmission can occur in a cell-to-cell manner or in a cell-free manner via virions. Cell-to-cell transmission (Fig. 1A) is commonly observed in diverse viruses such as paramyxoviruses, herpesviruses, and some retroviruses (Sattentau 2008). Some viruses can be transmitted from cell to cell without the participation of free viral particles. One mechanism includes fusion of the membranes of an infected and neighboring cell, resulting in the formation of multinucleated cells (syncytia), as is the case for paramyxovirus and herpesvirus. Other mechanisms include the formation of a motile surface extension (e.g. poxvirus) (Sattentau 2008). Some viruses such as HIV-1 can be transmitted in cell-to-cell manner through virological synapsis, which was estimated to account for more than 50 per cent of the total viral transmission under experimental conditions (Iwami et al. 2015). Cell-to-cell transmission can also be induced for viruses that are normally transmitted in a cell-free manner when blocking the protease activity that cleaves the bond between viral glycoprotein and the host receptor; this mechanism has been observed for influenza viruses in patients treated with oseltamivir (Tamiflu) (Mori, Haruyama, and Nagata 2011; Mori et al. 2015). Such cell-to-cell transmission could result in coinfection of the same cell with different viral variants or quasispecies, enabling their ‘cooperation’ (Ojosnegros et al. 2011; Shirogane, Watanabe, and Yanagi 2012; Bordería et al. 2015).

Cell-to-cell transmission allows a pair of viruses with resistance to a single drug to persist for a prolonged period of passages, whereas they would otherwise not be able to replicate on their own under double-drug treatment. Ultimately, the two viruses may recombine their independently emerged mutations or accumulate a second resistance mutation to generate a double-resistant virus. Thus, we here propose a hypothesis that the probability of developing double-drug resistance is much higher for viruses that spread by cell-to-cell transmission than for viruses that spread by cell-free transmission.

To test this hypothesis, we here theoretically examine the probability of the emergence of double-resistant viruses under two-drug treatment. First, we use a simple stochastic model describing the frequency changes of viral genotypes over successive infections based on the Wright–Fisher (WF) model (Crow and Kimura 1970) in which an infected cell always reproduces one infected cell. By using this model, we analytically demonstrate that the probability of a virus acquiring double resistance is approximately the square of the number of viral genomes that establish infection times greater in cell-to-cell transmission than in cell-free transmission. Second, the model is expanded to consider not only the frequency dynamics within infected cells but also the stochastic dynamics of the number of infected cells. We numerically calculate the emergence probability of double resistance in this expanded model and show that cell-to-cell transmission also causes greater emergence probability than cell-free transmission. These results suggest the importance of cell-to-cell transmission in the emergence of multidrug-resistant viruses.

## Model

### Assumptions

When a new infection occurs, one or more viral genomes enter into a target cell. We define $n$ as the number of viral genomes that establish infection, and it is assumed to be constant in all infected cells for simplicity. In the case of cell-to-cell transmission, these $n$ viral genomes originate from the same infected cell, but the frequencies of each viral genome in the newly infected cell are not always the same as in the donor-infected cell due to random sampling (Fig. 1B). In the following, we will model how the viral genome frequencies change through this random sampling of viral genomes. In the case of cell-free transmission, we assume that $n = 1$ (no coinfection occurs) because the density of virions per target cell would be so low that at most one virion can infect a target cell.

In this study, four types of viral genomes are considered: the wild-type ab, which is susceptible to both types of antiviral drugs; the single-resistant mutants Ab and aB, which are resistant to one of the drugs but susceptible to the other; and the double-resistant mutant AB, which is resistant to both drugs. Only infected cells containing both Ab and aB genomes (or containing AB genomes that do not exist initially) can survive under the simultaneous treatment of two drugs. We are interested in the emergence probability of the double-resistant virus, that is, the probability that AB genome is produced by mutation and is selected as one of the $n$ genomes that establish infection.

Figure 1C shows the schematic description of our model over successive infections. In an infected cell, each viral genome replicates and a genome pool for newly infected cells is produced (see $t$-th generation of infected cells in Fig. 1C). When a new infection occurs, $n$ viral genomes are randomly sampled from the genome pool of the present generation. Since we assume that there is no difference in the replication ability among genomes irrespective of whether they have drug resistance or not, the frequencies of genomes basically do not change within an infected cell. However, a viral genome in the genome pool may gain drug resistance by mutation with some probability (this happens in the genome pool of the $\left( {t - 1} \right)$-th generation in Fig. 1C) and we call this probability the mutation rate per generation, $u$. It should be noted that we assume no recombination between two loci that contribute the drug resistance. Moreover, we also assume that the mutation rates from a to A and b to B are the same for simplicity.

### WF model with killing to analyze the emergence probability of double-drug resistance

The process shown in Fig. 1C is called the WF model, where this model assumes that an infected cell in the current generation always produces one infected cell in the next generation before its natural death not due to drug treatment. Hence, the number of infected cells is not expanding nor shrinking over time. Since we are interested in the emergence probability of double resistance, the process is terminated (killed) when an AB genome appears by mutation and is selected as one of the $n$ genomes that establish infection. This model is called as the WF model with killing (Karlin and Taylor 1981; Karlin and Tavaré 1981a,b,c; Slatkin and Takahata 1985; Takahata and Slatkin 1986).

Here, we assume that the initial infected cell contains only two types of single-resistant genomes, Ab and aB, for simplicity, and the analysis including the wild-type ab is examined numerically later. Let $i$ be the number of Ab genomes that establishes infection, which means that $n - i$ other genomes that establish infection are of aB type. Note that $i$ should be $0 \lt i \lt n$; otherwise (i.e. if $i = 0\ {\mathrm{or}}\ n$), the infected cell cannot survive or reproduce under the double-drug treatment (indicating viral extinction in a host). In the infected cell, the frequency of Ab genome in its genome pool is ${p_i} = \left( {1 - u} \right)i/n$ and that of aB genome is ${q_i} = \left( {1 - u} \right)\left( {1 - i/n} \right)$ because $u$ is the probability of gaining another drug-resistant mutation per generation. Then, the transition probability ${P_{ij}}$ that the infected cell containing $j$ Ab and $n - j$ aB genomes is produced from the infected cell containing $i$ Ab and $n - i$ aB genomes is written as


(1) $$\begin{array}{*{20}{c}}
{\begin{array}{*{20}{c}}
{{P_{ij}} = \left( {\begin{array}{*{20}{c}}
n\\
j
\end{array}} \right){{\left( {{p_i}} \right)}^j}\,{{\left( {{q_i}} \right)}^{n - j}}}\\
{\;\;\;\;\;\;\;\;\;\;\;\;\;\;\;\;\;\;\;\;\;\;\;\;\;\;\;\;\;\;\;\;\; = \left( {\begin{array}{*{20}{c}}
n\\
j
\end{array}} \right)\,{{\left( {\frac{{{p_i}}}{{{p_i} + {q_i}}}} \right)}^j}{{\left( {\frac{{{q_i}}}{{{p_i} + {q_i}}}} \right)}^{n - j}}{{\left( {{p_i} + {q_i}} \right)}^n}.}
\end{array}}
\end{array}$$

The transition probability (1) does not include the possibility of the emergence of double resistance. Therefore, the emergence probability of double resistance per generation, or the probability that the WF process is ‘killed’, is


$$\begin{array}{*{20}{c}}
{\Pr \left[ {{\mathrm{process\,is\,killed}}} \right]\; = 1 - \mathop \sum \limits_{j = 0}^n {P_{ij}}}\\
{\;\;\;\;\;\;\;\;\;\;\;\;\;\;\;\;\;\;\;\;\;\;\;\;\;\;\;\;\;\;\;\;\;\;\; = 1 - {{\left( {{p_i} + {q_i}} \right)}^n}}\\
{\;\;\;\;\;\;\;\;\;\;\;\;\;\;\;\;\;\;\;\;\;\;\;\;\;\;\;\;\;\;\;\;\; = 1 - {{\left( {1 - u} \right)}^n}.}
\end{array}$$

According to these transition probability and killing probability, example trajectories of the frequency of Ab genome are written in Fig. 1B. In one case, an AB genome was generated by mutation and chosen as one of the genomes that establish infection, and then, the process is killed. In other cases, the Ab genome fixed or went extinct, which leads to the death of the infected cell under the double-drug treatment.

If ${\phi _i}$ denotes the probability that, staring with $i$ Ab and $n - i$ aB genomes, the population of viral genomes eventually goes to fixation of either Ab or aB genome (this leads to viral extinction due to the treatment) before the process is killed (i.e. before a double-resistant virus emerges), it satisfies


(2) $$\,\,\,\,\,\,\,\,\,\,\,\,\,\,\,\,\,\,\,\,\,\,\,\,\,\,{\phi _i} = \mathop \sum \limits_{j = 0}^n {P_{ij}}{\phi _j},\qquad \left( {i = 1,\,2, \cdots ,n - 1} \right),\,\,\,\,$$

with ${\phi _0} = {\phi _n} = 1$. The probability of the eventual emergence of an AB genome equals 1 (probability of the fixation of either Ab or aB genome), that is, $1 - {\phi _i}$.

### Diffusion process with killing

We further analyzed the process described earlier by diffusion approximation assuming that $n$ is sufficiently large. Let the frequency of the Ab type, ${X_t} = i/n$, be the state variable of this WF process at generation $t$. Given that the process is not killed, the number $j$ of Ab genomes in the next generation follows a binomial distribution with mean $n{p_i}/\left( {{p_i} + {q_i}} \right)$ and variance $n{p_i}{q_i}/{\left( {{p_i} + {q_i}} \right)^2}$. Hence, for $\delta X = {X_{t + 1}} - {X_t}$, its infinitesimal mean ${M_{\delta X}} = E\left[ {\delta X{\mathrm{|}}{X_t} = x} \right]$ and variance ${V_{\delta X}} = E\left[ {{{\left( {\delta X} \right)}^2}|{X_t} = x} \right]$, given that ${X_t} = i/n \equiv x,$ are


$$\begin{array}{*{20}{c}}
{{M_{\delta X}} = 0,}\\
{{V_{\delta X}} = \frac{{x\left( {1 - x} \right)}}{n}{{\left( {1 - u} \right)}^n}.}
\end{array}$$

When $u$ is very small, the killing probability $k = \Pr [{\mathrm{killed}}\,|{X_t} = x]$ is


$$k = 1 - {\left( {{p_i} + {q_i}} \right)^n} = 1 - {\left( {1 - u} \right)^n} \approx nu.$$

By introducing a new parameter


$$U = {n^2}u,$$

we obtain


$$\begin{array}{*{20}{c}}
{{M_{\delta X}} = 0,}\\
{{V_{\delta X}} = \frac{{x\left( {1 - x} \right)}}{n}{{\left( {1 - \frac{U}{{{n^2}}}} \right)}^n} = \frac{{x\left( {1 - x} \right)}}{n} + O\left( {\frac{1}{{{n^2}}}} \right),}\\
{k = 1 - {{\left( {1 - \frac{U}{{{n^2}}}} \right)}^n} = \frac{U}{n} + O\left( {\frac{1}{{{n^2}}}} \right).}
\end{array}$$

Therefore, by rescaling time as $\tau = t/n$ and taking the limit of large $n$, we derive the diffusion approximation for the fixation probability $\phi \left( x \right)$, where $x$ is the initial frequency of Ab genome, as follows:


(3) $$\begin{array}{*{20}{c}}
{\frac{{x\left( {1 - x} \right)}}{2}\phi {^{^{\prime} ^{\prime}}}\left( x \right) - U\phi \left( x \right) = 0,}
\end{array}$$

with $\phi \left( 0 \right) = \phi \left( 1 \right) = 1$, where $\phi {^{^{\prime}}}\left( x \right) = d\phi /dx$ and $\phi {^{^{\prime} ^{\prime}}}\left( x \right) = {\rm d}^{2}\phi /{\rm d}{x^2}$. This is a hypergeometric differential equation and can be solved analytically (the solution is shown in Appendix A in the Supplementary material). Therefore, the emergence probability of double resistance depends only on the parameter $U = {n^2}u$.

Notably, in the normal WF model, or the Moran model for population genetics, the effect of a mutation is measured by $nu$, the product of the effective population size and the mutation rate, which indicates the ratio of the mutation rate $u$ relative to the strength of random genetic drift $1/n$. Therefore, the expected time until the loss or fixation of a neutral viral allele with an initial frequency of 0.5 is $n$ generations (Crow and Kimura 1970). The intuitive reason why $U = {n^2}u$, not $nu$, determines the fixation probability (or 1—emergence probability) in our model is that the killing probability per generation is approximately $k = nu$ instead of $u$ in the normal WF model. Moreover, the equation for the fixation probability $\phi \left( x \right)$ in the normal WF model without killing contains only terms proportional to $\phi^{\prime\prime}\left( x \right)$, corresponding to the effect of random drift, and $\phi^{\prime}\left( x \right)$, corresponding to the directional change in gene frequency (by selection, mutation, and migration), whereas terms similar to $ - U\phi \left( x \right)$ are lacking. The terms proportional to $\phi \left( x \right)$ appear in the model with local extinction and colonization in a metapopulation (Boorman and Levitt 1973; Kimura 1983).

### Stochastic process of infected cell population

The WF model assumes that an infected cell always reproduces one new infected cell throughout its life. However, in the host body, we should consider the stochasticity of the number of new infections caused by an infected cell because the infected cell that includes both Ab and aB genomes should be rare. Suppose that the rate of causing a new infection and the rate of natural death of an infected cell per unit time (e.g. per day) are $r$ and $d$, respectively. Then, the number of infected cells is written by a branching process, and the number of infections caused by an infected cell before it dies, $l$, is written by a geometric distribution,


$$\,\,\,\,\,\,\,\,\,\,\,\,\,\,\Pr \left( {Y = l} \right) = w{\left( {1 - w} \right)^l},\,\,\,\,\,\,\,\,\,\,\,\,\,\left( {l = 0,1, \ldots } \right)$$

where $w = d/\left( {r + d} \right)$. The mean number of secondary infected cells is


$$m = E\left[ l \right] = \frac{{1 - w}}{w} = \frac{r}{d}.$$

We combine this branching process with the WF model with killing, assuming a constant $n$. Let ${\psi _i}$ denote the probability that the descendants of an infected cell carrying $i$ Ab and $n - i$ aB genomes never experience the emergence of double-resistant AB. Then, ${\psi _i}$ satisfies the following equation:


(4) $$\begin{array}{*{20}{c}}
\begin{array}{l}
\,{\psi _i}\; = \mathop \sum \limits_{l = 0}^\infty w{\left( {1 - w} \right)^l}{\left( {\mathop \sum \limits_{j = 0}^n {P_{ij}}{\psi _j}} \right)^l}\\
\;\;\;\; = \frac{w}{{1 - \left( {1 - w} \right)\mathop \sum \nolimits_{j = 0}^n {P_{ij}}{\psi _j}}}\,\\
\;\;\;\; = \frac{1}{{1 + m\left( {1 - \mathop \sum \nolimits_{j = 0}^n {P_{ij}}{\psi _j}} \right)}},\,\,\,\,\,\,\,\,\,\,\,\,\,\,\,\left( {i = 1,2, \ldots ,n - 1} \right)
\end{array}
\end{array}$$

with ${\psi _0} = {\psi _n} = 1$, and ${P_{ij}}$, the transition probability that the number of Ab genomes changes from $i$ to $j$, is defined in equation (1). This equation is approximated as


$${\psi _i} \approx 1 - m\left( {1 - \mathop \sum \limits_{j = 0}^n {P_{ij}}{\psi _j}} \right),$$

when $\mathop \sum \limits_{j = 0}^n {P_{ij}}{\psi _j} \approx 1$, which means that double resistance hardly emerges. Moreover, if we assume that the mean number of secondary infected cells is 1 ($m = 1$), this equation is completely in accordance with equation (2).

### Monte Carlo simulation of infected cell population growth

The dynamics of the infected cell population were simulated by the birth–death process starting with one infected cell, which has the same number ($n/2$) of Ab and aB genomes. Each infected cell has a probability of death $w = 1/\left( {1 + m} \right)$, as defined in the stochastic process of infected cell population. For each iteration, one cell is randomly selected, which dies with probability $w$; otherwise, it will produce a newly infected cell. The number of Ab genomes in the newly infected cell (denoted by $j$) produced from a donor cell including $i$ Ab genomes is randomly assigned according to the transition probability (1). If the newly produced infected cell has $n$ or no Ab genomes, it will die immediately because the presence of both Ab and aB genomes is required for survival under double-drug treatment. For each reproduction event, the probability of the emergence of double resistance is $1 - {\left( {1 - u} \right)^n}$. The simulation ends when double resistance emerges or the infected cell population goes to extinction.

## Results

### Analytical results

The solution to (3) is obtained (see Appendix A in the Supplementary material) as


(5) $$\begin{array}{*{20}{c}}
{\phi \left( x \right) = A\left[ {1 + \mathop \sum \limits_{n = 1}^\infty \frac{{\mathop \prod \nolimits_{j = 1}^n \left( {\left( {j - 1} \right)\left( {2j - 3} \right) + U} \right)}}{{\left( {2n - 1} \right)!!}}\frac{{{{\left( {2x - 1} \right)}^{2n}}}}{{n!}}} \right]}
\end{array}$$

with


$$A = \frac{1}{{\sqrt \pi }}{\Gamma}\left( {\frac{{3 - \sqrt {1 - 8U} }}{4}} \right){\Gamma}\left( {\frac{{3 + \sqrt {1 - 8U} }}{4}} \right)$$

where $\left( {2n - 1} \right)!! = \left( {2n - 1} \right)\left( {2n - 3} \right) \cdots 3 \cdot 1$ and ${\Gamma}\left( z \right)$ is the Gamma function. As is clear from the form of each term in (5), the fixation probability is at the minimum at the initial frequency $x = 0.5$:


$$\begin{array}{*{20}{c}}
{\mathop {\min }\limits_x \phi \left( x \right) = \phi \left( {\frac{1}{2}} \right) = A}\\
{\;\;\;\;\;\;\;\;\;\;\;\;\;\;\;\;\;\;\;\;\;\;\;\;\;\;\;\;\;\; = 1 - \left( {2\ln 2} \right)U + O\left( {{U^2}} \right)}
\end{array}$$

**Figure 2.**
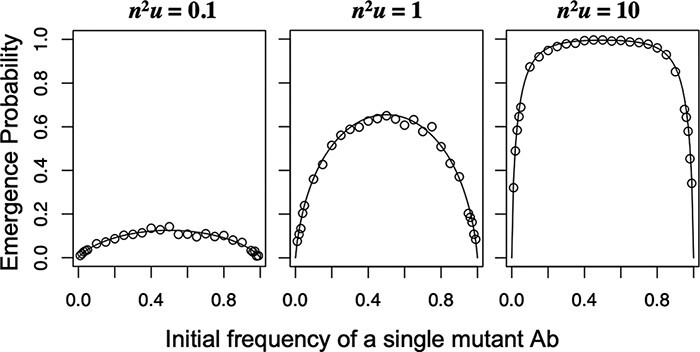
The emergence probability of double resistance as a function of the initial frequency of single-resistant viral genomes. When the initial frequency of a single-mutant Ab is $x$, the initial frequency of the other single-mutant aB is $1 - x$. $U = {n^2}u = $ 0.1, 1, and 10 from the left to right. Dots indicate the fraction of the emergence of double resistance in 1,000 independent runs of Monte Carlo simulations with a multiplicity of infection of 100, and lines indicate the analytical results.

**Figure 3.**
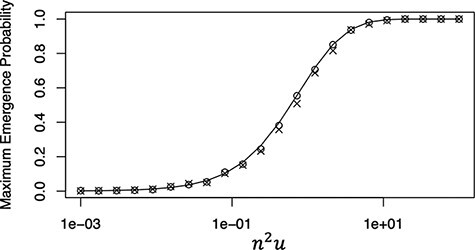
Emergence probability of double resistance plotted against ${n^2}$ times the mutation rate. The initial frequencies of single mutants are assumed to be equal ($x = 0.5$). Monte Carlo simulation results are the fraction of the emergence of double resistance in 1,000 independent runs for $n = 100$ (open circles) and $n = 10$ (cross-hatched), respectively. The solid line represents the analytic result calculated using equation (5).

Therefore, the emergence probability of double resistance for cell-to-cell transmission, $P_{{\mathrm{emerge}}}^{{\mathrm{CTC}}}\left( x \right) = 1 - \phi \left( x \right)$, has the maximum


$$P_{{\mathrm{emerge}}}^{{\mathrm{CTC}}}\left( {\frac{1}{2}} \right) = \left( {2\ln 2} \right)U + O\left( {{U^2}} \right)$$

where $U = {n^2}u$, $n$ is the number of viral genomes that establish infection, and $u$ is the rate of mutation at which double resistance emerges per generation. This analytical result showed good consistency with the results of the Monte Carlo simulation (Figs 2 and 3). Moreover, the simulations further confirmed that the emergence probability is determined by a single parameter: ${n^2}u$ (Fig. 3).

### Effect of cell-to-cell transmission

If there is no cell-to-cell transmission, the emergence probability of double resistance is $u$ (note that $n = 1$)


$$P_{{\mathrm{emerge}}}^{{\mathrm{CF}}} = u$$

**Figure 4.**
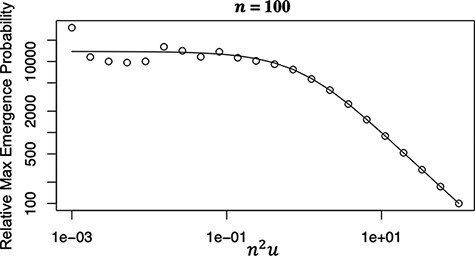
Effect of cell-to-cell transmission on promotion of the emergence of double resistance. The odds ratio for the emergence probability of double resistance in cell-to-cell transmitted viruses to that in cell-free transmitted viruses is plotted as a function of $U = {n^2}u$, $n = 100$, and hence, the odds ratio is approximately 13,900 for small $U$ ($U$ smaller than 0.1). Open circles show the results of Monte Carlo simulations, and the solid line represents the analytical formula $r_{{\mathrm{CF}}}^{{\mathrm{CTC}}} = \left( {1 - A} \right)/u = \left[ {1 - {{\left( \pi \right)}^{ - \frac{1}{2}}}{\Gamma}\left( {\frac{{3 - \sqrt {1 - 8U} }}{4}} \right){\Gamma}\left( {\frac{{3 + \sqrt {1 - 8U} }}{4}} \right)} \right]/u$, which tends to $\left( {2\ln 2} \right){n^2}$, in this case, $1.39 \times {10^4}$ for small $U$.


Figure 4 shows the emergence probability of double resistance with cell-to-cell transmission relative to that for cell-free transmission only,

$$\frac{{P_{{\mathrm{emerge}}}^{{\mathrm{CTC}}}\left( {\frac{1}{2}} \right)}}{{P_{{\mathrm{emerge}}}^{{\mathrm{CF}}}}} \approx \left( {2\ln 2} \right){n^2} = 1.39 \times {n^2}$$

for small $U = {n^2}u$. Therefore, the extent to which cell-to-cell transmission promotes the emergence of double resistance is $1.4 \times {n^2}$ times larger than that of cell-free transmission. In other words, if $n = 10$, the emergence is 140 times more likely for viruses with cell-to-cell transmission than for viruses with cell-free transmission only; if $n = 100$, it is 14,000 times more likely.

### Initial population with the wild-type genotype

**Figure 5.**
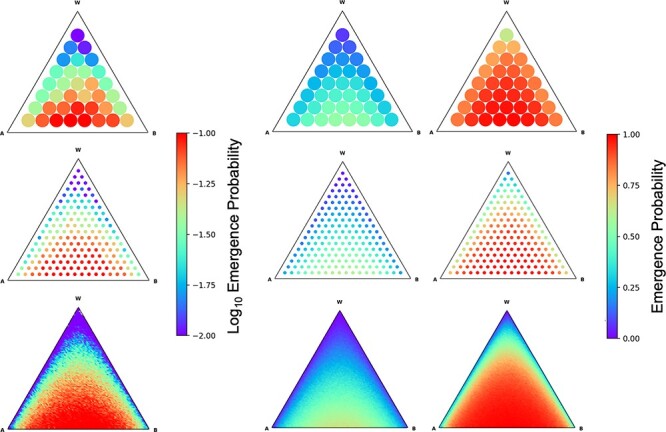
Emergence probability of double resistance against the initial frequencies of wild-type and single mutants. The colors of the points show the emergence probability in the simulations. The position of point in the triangle (simplex) indicates the initial frequencies of Ab (lower left direction), aB (lower right direction), and wild-type (upper direction) viruses, and hence, the results on the bottom edge in each simplex correspond to the results when the initial population consists only of the single-resistant Ab and aB (Fig. 2). The difference between each simplex is that $U = {n^2}u = $ 0.1, 1, and 10 from the left to right columns, and $n = $ 10, 100, and 1000 from the top to bottom rows.

For the sake of mathematical tractability, we have so far assumed that the initial infected cell contains only Ab and aB genomes (i.e. the two kinds of single-resistant viral genomes without the wild-type ab). Here, we examine how this simplifying assumption affects the results by conducting Monte Carlo simulations of the same process including the wild type. As the wild type does not have resistance to either drug, its presence does not affect the survivorship of an infected cell, that is, viral extinction in a host occurs once either Ab or aB genome is lost. However, in this case, random sampling is derived from three rather than two genotypes, and thus, the probability that the process is killed changes. In Fig. 5, each point in the triangle indicates the initial frequencies of Ab, aB, and wild type. The colors of the points show the proportion of emergence of AB in 1,000 runs of Monte Carlo simulations, and each column indicates the results for different $U = {n^2}u$. In general, a double-resistant AB is more likely to emerge when both Ab and aB genomes are present at similar frequencies than when either dominates. This tendency is consistent with the results shown in Fig. 2. Moreover, the initial condition dependencies are similar between rows in the same column, indicating that the ${n^2}u$ scaling of the emergence probability is robust even when allowing wild-type viruses to segregate initially.

### Effect of cell population dynamics

The dynamics of the infected cell population are characterized by the mean number of new infections from an infected cell, $m$. Here, we consider three cases: steady, shrinking, and expanding cell populations.

#### Emergence of double resistance in a steady infected cell population

**Figure 6.**
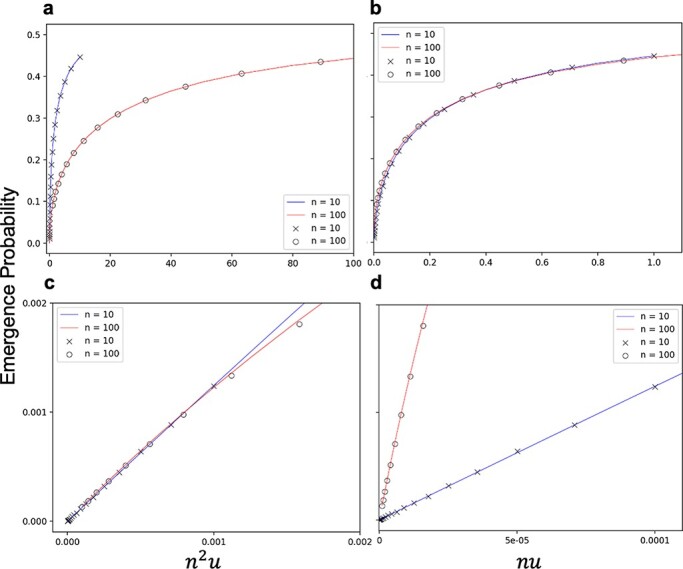
Emergence probability in a steady infected cell population. In all cases, the mean number of new infections from an infected cell $m$ is 1, and the initial frequency of a single mutant is set to 0.5. Monte Carlo simulation results of ${10^7}$ independent runs are shown by open circles ($n = 100$) and cross-hatchings ($n = 10$), respectively. The solid line shows the numerical solution of equation (4). (A, C) Emergence probability is plotted against ${n^2}u$. The results are obtained with large $u$ for (A) and small $u$ for (C), and therefore, (C) shows a magnified view of a very small range of ${n^2}u$ in (A). (B, D) Emergence probability is plotted against $nu$. The ranges of $u$ for (B) and (D) are the same as in (A) and (C), respectively.

This case corresponds to chronic infection or a viral infection maintained at a set point, in which the deaths of infected cells are in balance with the birth of newly infected cells. Since the WF model can be regarded as a reflection of cell dynamics with $m = 1$, we expected the emergence probabilities with $m = 1$ to be consistent with those in the WF model. Figure 6 shows the dependence of the emergence probability obtained by the numerical solution of (4) and the Monte Carlo simulation, where the initial frequency of the single-resistant virus was set to 0.5 for each genotype. The emergence probability of double resistance is determined by $nu$ if $u$ was sufficiently large (compare Fig. 6A and B), whereas it is determined by ${n^2}u$ if $u$ was sufficiently small (see Fig. 6C and D). In other words, two scaling schemes determine the emergence probability of double resistance. The ${n^2}u$ scaling scheme is consistent with the results of the WF model. This result can be explained by the fact that equation (4) can be approximated by equation (2) when $u$ is very small; in such a case, the Taylor expansion for very small ${\psi _i}$s ($i = 1,2, \cdots ,n - 1$) in (4) leads to (2). This is also confirmed by comparing the results of the Monte Carlo simulation with the expectation given by the diffusion approximation (Supplementary Figure S1).

**Figure 7.**
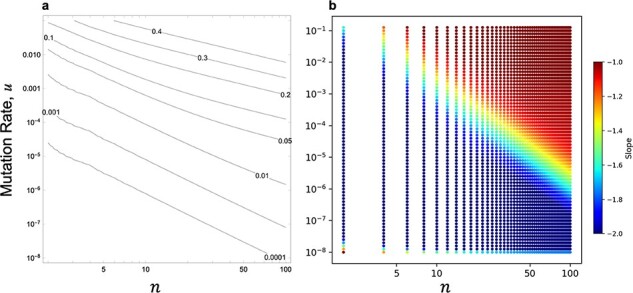
Parameter dependence and scaling schemes of emergence probability in a steady infected cell population. (A) Contour plot of the emergence probability. (B) The slopes of the contours in the $n$–$u$ plane (for the calculation of slopes, see the Methods section). The emergence probability is calculated by the numerical solution of equation (4) with $m = 1$, and the initial frequency of a single mutant is set to 0.5.

The two scaling schemes is also seen in the contour plot of the emergence probability in the $n$–$u$ plane (Fig. 7A). The scaling scheme is evaluated by the slopes of the contours in Fig. 7A, and they are close to 1 ($nu$ scaling) at the upper right (large $u$) but become close to 2 (${n^2}u$ scaling) at the lower left (small $u$) (Fig. 7B). It should be noted that the intermediate value of the slope is seen in only a limited region, which means that the scaling scheme is either $nu$ or ${n^2}u$ in the majority of the region, and the border of the scaling scheme is given approximately as $u = \left( {{\mathrm{const}}} \right) \times {n^{ - 3}}$ (the dividing line between two scaling schemes in Fig. 7B in the log–log plot for $n$ and $u$ had a slope of approximately $ - 3$, giving rise to the relationship $u = \left( {{\mathrm{const}}} \right) \times {n^{ - 3}}$ for the boundary).

The ${n^2}u$ scaling in the emergence probability of double resistance can be understood by the same reason as in the WF model with killing, that is, (1) the killing rate per generation is approximately $nu$ and (2) the stochastic loss of either Ab or aB genome occurs approximately in $n$ generations. Therefore, we hypothesized that by changing the second point (e.g. the number of generations for an infected cell line does not depend on $n$), the emergence probability will shift to an $nu$ scaling pattern. To confirm this hypothesis, we calculated the emergence probability without frequency dynamics of single-resistant genomes in an infected cell (Appendix B in the Supplementary material). Specifically, we considered the case where no death of infected cells occurs due to the loss of either single-resistant genomes, so that the population dynamics of infected cells is independent of $n$. Under such an assumption, we confirmed that the emergence probability is very close to the region of $nu$ scaling in Fig. 7A (upper right of Supplementary Figure S2).

In the case of cell-free transmission with $m = 1$, the emergence probability of double resistance is $u$, assuming that there is one infected cell at the start of the double-drug treatment. Therefore, we can conclude that cell-to-cell transmission promotes the emergence probability of double resistance by ${n^2}$ times compared to the emergence probability with cell-free transmission in the region of ${n^2}u$ scaling, while the amplification factor is $n$ in the region of $nu$ scaling.

#### Emergence of double resistance in shrinking and expanding infected cell populations

**Figure 8.**
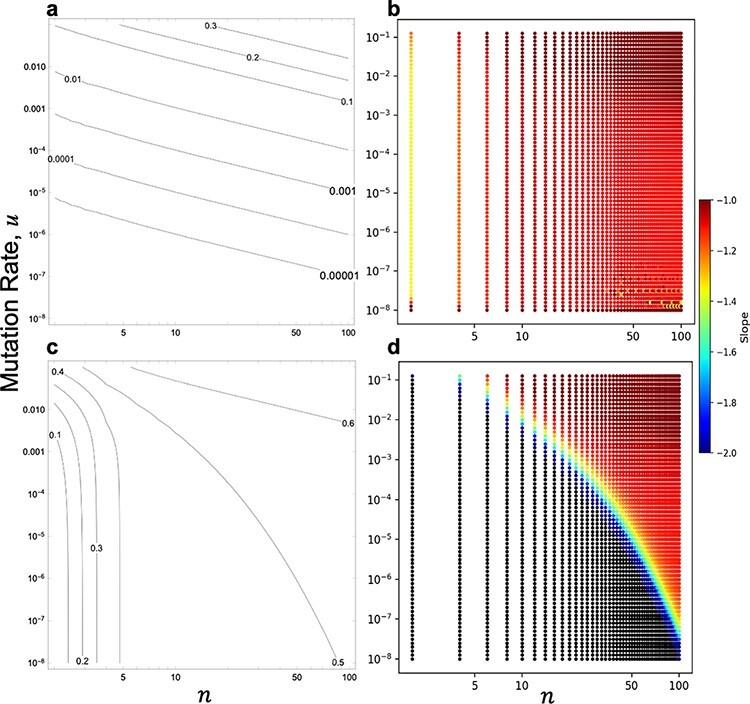
Parameter dependence and scaling schemes of emergence probability in shrinking and expanding infected cell populations. The contour plot of the emergence probability (A) and the slopes of the contours (B) in the $n$–$u$ plane for a shrinking population ($m = 0.5$). The contour plot of the emergence probability (C) and the slopes of the contours (D) in the $n$–$u$ plane for an expanding population ($m = 2$), where the points with a slope lower than $ - 2$ are shown in black in (D). In all cases, the initial frequency of a single mutant is set to 0.5.

**Figure 9.**
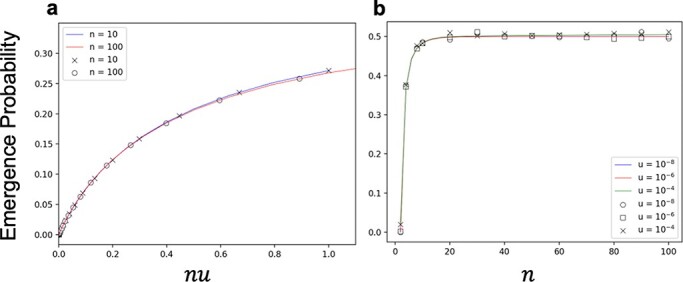
Comparison of simulation with the analytical solution in shrinking and expanding infected cell populations. The symbols indicate the results of Monte Carlo simulation, and solid lines indicate the numerical solution of equation (4). In all cases, the initial frequency of the single mutant is set to 0.5. (A) Emergence probability against $nu$ for $m = 0.5$. The results are calculated with $n = 100$ (open circles and red line) and $n = 10$ (cross-hatching and blue line). (B) Emergence probability against $n$ for $m = 2$. The results are calculated with $u = {10^{ - 8}}$ (open circles and blue line), $u = {10^{ - 6}}$ (open squares and red line), and $u = {10^{ - 4}}$ (cross-hatching and green line).

A shrinking infected cell population corresponds to $m \lt 1$, whereas an expanding infected cell population corresponds to $m \gt 1$. The emergence probability could also be numerically calculated for these situations from equation (4). When $m = 0.5$, the emergence probability showed $nu$ scaling in the majority of the parameter range examined (Fig. 8A and B). This result suggests that the infected cell population is likely to go extinct because of the low $m$, and the fixation of either single mutant has little effect. When $m = 2$, $nu$ scaling is limited to large $u$ and $n$ (upper right in Fig. 8D), as in the case with $m = 1$. In another region, the death of infected cells by fixation of a single mutant clearly affects the emergence probability. In particular, if $n$ is very small, the emergence probability depends only on $n$ (lower left in Fig. 8C) because such emergence fails only when the infected cell population becomes fixed to one of the single mutants in the early stage of population dynamics. The results of the Monte Carlo simulation for $m = 0.5$ and $m = 2$ are shown in Fig. 9. For both cases, the results of the simulation were consistent with the numerical solution of equation (4). Since $m = 1$ is the special case, we also compared the results with $m$ close to one. As $m$ becomes closer to 1, the result seems to converge to that of $m = 1$ (Supplementary Figures S3 and S4). These results suggest that ${n^2}u$ scaling appears when $m$ is sufficiently close to one and $n$ and $u$ are sufficiently small.

## Discussion

The emergence of drug resistance is one of the most serious contemporary public health issues. For example, the emergence of Tamiflu-resistant influenza virus was reported only a few years after the introduction of Tamiflu to the market. As Tamiflu inhibits the release of virions from an infected cell by blocking the protease activity of neuraminidase, it could efficiently block the transmissibility of influenza viruses, which are known to spread exclusively through cell-free infection. However, the block of protease activity to cleave the binding between the host receptor and viral hemagglutinin has been found to cause a shift to cell-to-cell transmission of viruses (Mori, Haruyama, and Nagata 2011), accompanied by the accumulation of viral mutants in the cell-to-cell-infected cells (Mori et al. 2015). Our study was motivated by this drug-induced cell-to-cell transmission in influenza viruses. We considered that the mass transmission of heterogeneous viral genomes from the donor infected cell to the recipient cell in cell-to-cell transmission would allow the mixture of a pair of single mutants to survive, which cannot survive if either is missing. It is well known that a mutant viral genome that encodes a defective protein can survive through the expression of a corresponding normal protein by a wild-type genome (Domingo, Sheldon, and Perales 2012). This type of interaction is known as complementation, and many such cases have been reported both *in vitro* and *in vivo* (natural infections) (Moreno et al. 1997; Aaskov et al. 2006; Gelderblom et al. 2008). In these cases, the mutant genome tends to be a ‘free rider’; however, there is also an example of mutual complementation. Ojosnegros et al. (2011) reported the emergence of two defective genomes of the foot-and-mouth disease virus complementing each other. These genomes have deletions in different essential genes, but could replicate by coinfecting the same cell. In line with our hypothesis, our model clearly showed that cell-to-cell transmission can accelerate the speed of emergence of a virus with a selective advantage only after multiple necessary mutations are accumulated in the wild type. This is because the virus has the opportunity to wait for further mutations in either single mutant to occur to produce another mutation before the loss of either single mutant causes viral extinction.

Our model further suggests that the emergence probability depends only on ${n^2}u$, where $n$ is the number of viral genomes that establish infection and $u$ is the mutation rate of drug resistance per generation. This dependence is considered to be due to the emergence probability per one infection of $1 - {\left( {1 - u} \right)^n}\~nu$ and that the successive infection continues for $n$ generations on average. In contrast, the emergence of double resistance under two-drug treatment for a single-resistant virus that spreads through cell-free transmission can only occur via replication; thus, the emergence probability is $u$. If ${n^2}u$ is sufficiently small, the emergence probability becomes proportional to ${n^2}u$, which is shown by the diffusion approximation. Hence, cell-to-cell transmission has ${n^2}$ times the emergence probability of double resistance compared to cell-free transmission. Although this comparison is based on our assumption that all $n$ viral genomes in an infected cell have either drug-resistant mutation in the case of cell-to-cell transmission, we can also relax this assumption and allow wild-type viruses to segregate initially. Indeed, the results of the Monte Carlo simulation suggested that the ${n^2}u$ scaling of emergence probability still occurs under this relaxed assumption (Fig. 5).

We also extended the model to include the stochastic dynamics of infected cells. In this extended model, the dynamics of infected cells are characterized by the parameter $m$ that denotes the mean number of new infections from an infected cell. When $m = 1$, the infected cell population is expected to neither expand nor shrink. This would be approximately expected when the death rate of infected cells under an immune response is balanced with the birth rate of newly infected cells, which is expected for viruses that are maintained at a set point in the case of chronic infection. Since this situation matches the assumption of the WF model, the emergence probability of the cell dynamics model becomes consistent with that of the WF model for the parameter regions in which the mean number $nu$ of mutations per infection is small (lower left half of Fig. 7B). This suggests that we can use the analytical results from equation (5) even if explicitly taking into account the dynamics of infected cells in such regions. Such situations are likely to not be rare in reality, because it is natural to assume that not only the mutation rate $u$ but also the $n$ of cell-to-cell transmission is small, at least for some viruses. Although millions of viral genomes are known to spread by cell-to-cell transmission, the number of genomes that contribute to viral replication would be limited and the $n$ in our model is the latter case. In fact, Miyashita and Kishino (2010) estimated that the number of genomes establishing infection in adjacent cells via cell-to-cell transmission for *Soil-borne wheat mosaic virus* is approximately five.

When comparing the total risk of the emergence of double-drug resistance under cell-to-cell transmission with that of cell-free transmission, it is essential to know the frequency of drug-resistant viruses before treatment and whether an infected cell harbors two kinds of single-resistant viruses. The frequency of drug-resistant viruses before treatment has been discussed in the case of cell-free transmission (Ribeiro, Bonhoeffer, and Nowak 1998), which is proportional to $u/s$—where $u$ is the mutation rate and $s$ ($0 \lt s \lt 1$) is the selective disadvantage of the mutant against the wild type before treatment—for a single-resistant virus. Since $n$ viral genomes are included in the case of cell-to-cell transmission, the probability that an infected cell has at least one single drug-resistant genome is roughly $n$ times larger than that in the case of cell-free transmission. Moreover, there is a possibility that an infected cell includes both single drug-resistant genomes, which is the situation considered in our present study. Taken together, we conclude that the risk of emergence of double-drug resistance will be higher under cell-to-cell transmission than cell-free transmission. The multidimensional diffusion model (Durrett 2008) may be useful in considering the probability that an infected cell harbors two kinds of single-resistant viruses before treatment and determining their frequencies, which remains an area of future work.

From a more general point of view, our model suggests that cell-to-cell transmission promotes viral evolution. Two examples support this suggestion. Mori et al. (2015) provided experimental support for this effect with two different temperature-sensitive mutants of influenza virus. Each of these mutant viruses could only survive when they coinfected the same cell at a non-permissive temperature. Since oseltamivir prevents cell-free transmission of influenza virus but promotes its cell-to-cell transmission (Mori, Haruyama, and Nagata 2011), the usage of oseltamivir rescued the increase of the temperature-sensitive mutants at a non-permissive temperature. Moreover, they showed that the serial passages of the wild-type virus in the presence of oseltamivir resulted in the faster accumulation of mutations in viral genomes than that observed in the absence of oseltamivir. Another example is provided by the measles virus, which can transfer its genome directly to adjacent cells through cell-to-cell membrane fusion. Shirogane, Watanabe, and Yanagi (2012) showed that co-expression of the wild-type and G264R-mutant fusion proteins of measles virus exhibited high fusogenic function. They also showed that the recombinant measles virus possessing both the wild-type and mutant fusion proteins efficiently spread in the hamster brain in contrast to the wild-type virus. This result indicates that coinfection of wild-type and mutant viruses could change the viral tissue tropism and produce a new phenotype.

In some viruses such as measles virus (Rager 2002) and Ebola virus (Beniac et al. 2012), multiple genomes have been observed in a single virus particle under experimental infection conditions. Moreover, Luque et al. (2009) observed this ‘polyploidy’ in the virions of the infectious bursal disease virus purified from natural populations. Since our model can be applied to polyploidy without additional assumptions, it will be important to examine how universal polyploidy is in viral families. Our result of the rapid emergence of double resistance under cell-to-cell transmission also suggests that viral ‘polyploidy’ could promote such evolution and, more generally, a similar evolutionary circumstance in which a virus needs to accumulate multiple mutations to increase its fitness.

There are several potential limitations to our model. First, we assume that an infected cell has full resistance to a drug if at least one drug-resistant viral genome exists in an infected cell. In reality, the level of resistance may correlate with the proportion of resistant viral genomes in an infected cell. In this case, the probability that an infected cell die due to the drug treatment will increase, and our estimation of the emergence probability of double resistance would thus represent overestimation. Second, we have not considered the target cell limitation of viral growth in our model. However, a low number of susceptible cells due to the spread of infection would correspond to a low value of $m$ (i.e. the mean number of new infections from an infected cell), although this is assumed to be constant in our model. Further analyses including dynamic feedback in the viral growth rate $m$ (e.g. the target cell limitation) would be an interesting future endeavor. Third, many viruses can use both cell-free transmission and cell-to-cell transmission. In such viruses, the emergence probability of double-drug resistance would vary from our present predictions. This is another direction for future study. Despite these limitations, our model demonstrates the importance of cell-to-cell transmission in the emergence of multiple drug-resistant viruses. This work suggests that finding a strategy to inhibit cell-to-cell transmission during multidrug treatment may help to reduce the risk of drug resistance and consequent treatment failure.

## Methods

### Calculation of the slope of contours of emergence probability in parameter space

Suppose the emergence probability for $k$ types of different $n$ (${n_1},\,{n_2},\, \ldots ,\,{n_k}$) and $l$ types of different $u$ (${u_1},\,{u_2},\, \ldots ,\,{u_l}$) in the $n$–$u$ plane. Let ${x_{ij}}$ be the emergence probability for ${n_i}$ and ${u_j}$. The slope of contour at $\left( {n,\,u} \right) = \left( {{n_i},\,{u_j}} \right)$, ${s_{ij}}$, is obtained by


$${s_{ij}} = \frac{{{u_j} - u_{i + 1,j}^*}}{{{n_i} - {n_{i + 1}}}},$$

where $u_{i + 1,j}^*$ is calculated by interpolation


$$u_{i + 1,j}^* = \frac{{\left( {{u_j} - {u_{j - 1}}} \right)\left( {{x_{ij}} - {x_{i + 1,j}}} \right)}}{{{x_{i + 1,j}} - {x_{i + 1,j - 1}}}} + {u_j},$$

if ${x_{ij}} \gt {x_{i + 1,1}}$. Otherwise, $u_{i + 1,j}^*$ is calculated by extrapolation,


$$u_{i + 1,j}^* = \frac{{\left( {{u_1} - {u_2}} \right)\left( {{x_{ij}} - {x_{i + 1,1}}} \right)}}{{{x_{i + 1,1}} - {x_{i + 1,2}}}} + {u_j}.$$

## Supplementary Material

vead017_Supp

## Data Availability

The statement of data availability is not applicable.
