# Supplementary material for: Cell-to-cell transmission promotes the emergence of double-drug resistance
Source: Virus Evol. 2023 Mar 11;9(1):vead017. doi: 10.1093/ve/vead017 (PMC10517696; doi:10.1093/ve/vead017)
Supplement: vead017_Supp [file vead017_supp.zip › Supplementary_Material.docx]

# Appendices

## Appendix A: Analytic expression for the fixation probability

The fixation probability $\phi(x)$ of either single-resistant genome over successive infections with the initial frequency $x$ satisfies

$$x\left( 1-x \right)\frac{d^{2}\phi}{dx^{2}}-2\mu\phi=0, (A1)$$

where $\mu=n^{2}u$, $n$ is the integer representing the number of viral genomes that establish infection, and $u$ is the rate at which another drug-resistant mutation occurs in a single-resistant genome per generation. The boundary condition is $\phi\left( 0 \right)=\phi\left( 1 \right)=1$. The emergence probability of double resistance before one of the single-resistant genome goes fixation (or one of the single-resistant genome goes extinct) is given by $P_{E}=1-\phi$.

The equation (A1) is the form of a hypergeometric differential equation, $x\left( 1-x \right)\phi''+\left( c-\left( a+b+1 \right)x \right)\phi'-ab\phi=0$, and can be solved by using hypergeometric functions [1]. However, to avoid the complicated forms of solutions for the cases for a non-positive integer c (actually in this case $c=0$), we transform the equation as in the following.

For simplicity, we transform the variables in two steps. We first change the variable as $y=2x-1$ with which $d/{dx}=2\left( d/{dy} \right)$, ${d^{2}}/{dx^{2}}=4\left( {d^{2}}/{dy^{2}} \right)$ and $x\left( 1-x \right)=\left( 1-y^{2} \right)/4$. The differential equation for $f\left( y \right)=\phi\left( x \right)$ corresponding to (A1) is then

$$\left( 1-y^{2} \right)\frac{d^{2}f}{dy^{2}}-2\mu f=0, f\left( -1 \right)=f\left( 1 \right)=1. (A2)$$

We then transform the variable as $z=y^{2}$. By noting

$$\frac{d}{dy}=\frac{dz}{dy}\frac{d}{dz}=2y\frac{d}{dz}=2\sqrt{z}\frac{d}{dz}$$

$$\frac{d^{2}}{dy^{2}}=\frac{d}{dy}\left( 2y\frac{d}{dz} \right)=2\frac{d}{dz}+4y^{2}\frac{d^{2}}{dz^{2}}=2\frac{d}{dz}+4z\frac{d^{2}}{dz^{2}},$$

we have the differential equation for $g\left( z \right)=f\left( y^{2} \right)$:

$$\left( 1-z \right)\left( 4z\frac{d^{2}}{dz^{2}}+2\frac{d}{dz} \right)g-2\mu g=0,$$

or

$$z\left( 1-z \right)\frac{d^{2}g}{dz^{2}}+\frac{1}{2}\left( 1-z \right)\frac{dg}{dz}-\frac{\mu}{2}g=0, (A3)$$

which have three regular singular points at $z=0$, $z=1$ and $z=\infty$. As we are interested in the fixation probability around $x=1/2$, we consider the solution around $z=0$ (corresponding to $x=1/2$). The boundary condition for (A3) is $g\left( 1 \right)=1$, as both $x=0$ and $x=1$ correspond to $z=1$.

The equation (A3) is also a hypergeometric differential equation of the form

$$z\left( 1-z \right)\frac{d^{2}g}{dz^{2}}+\left[ \gamma-\left( \alpha+\beta+1 \right)z \right]\frac{dg}{dz}-\alpha\beta g=0, (A4)$$

with $\gamma=1/2$, $\alpha+\beta+1=1/2$, and $\alpha\beta=\mu/2$. The parameters $\alpha$ and $\beta$ are obtained as $\alpha=-\left( 1-\theta\right)/4$, $\beta=-\left( 1+\theta\right)/4$ with $\theta=\sqrt{1-8\mu}$. The $\theta$ can be a complex number if $\mu>1/8$, but this does not make any problem as shown below. Because $\gamma$ is not an integer, two independent solution of A3 around $z=0$ are (15.5.3 and 15.5.4 of Abramowitz and Stegun [1]):

$$g_{1}\left( z \right)=F\left( \alpha, \beta;\gamma;z \right),$$

$$g_{2}\left( z \right)=z^{1-\gamma}F\left( \alpha-\gamma+1, \beta-\gamma+1;2-\gamma;z \right)$$

where

$$F\left( \alpha, \beta;\gamma;z \right)=\sum_{n=0}^{\infty} \frac{\left( \alpha\right)_{n}\left( \beta\right)_{n}}{\left( \gamma\right)_{n}}\frac{z^{n}}{n!}$$

is the hypergeometric function, with $\left( q \right)_{n}=q\left( q+1 \right)\left( q+2 \right)\cdots\left( q+n-1 \right)$ referring to the Pochhammer symbol.

As the second independent solution for $\gamma=1/2$,

$$g_{2}\left( z \right)=z^{1/2}F\left( \alpha+\frac{1}{2}, \beta+\frac{1}{2}; \frac{3}{2}; z \right)$$

is not differentiable at $z=0$ or at the intermediate gene frequency $x=1/2$, we can exclude this function from the basis constituting the solution to the diffusion equation (A1). Therefore, the general solution to (A3) is, with a positive constant A,

$$g\left( z \right)=AF\left( -\frac{1-\theta}{4},-\frac{1+\theta}{4}; \frac{1}{2}; z \right). (A5)$$

The unknown constant A is determined from the boundary condition $g\left( 1 \right)=1$. Noting the formula $F\left( a,b;c;1 \right)=\Gamma\left( c \right)\Gamma\left( c-a-b \right)/\Gamma\left( c-a \right)\Gamma\left( c-b \right)$ (15.1.20 of Abramowitz and Stegun [1], where $\Gamma\left( z \right)$ is the Gamma function),

$$g\left( 1 \right)=A\frac{\Gamma\left( \frac{1}{2} \right)\Gamma\left( \frac{1}{2}+\frac{1-\theta}{4}+\frac{1+\theta}{4} \right)}{\Gamma\left( \frac{1}{2}+\frac{1-\theta}{4} \right)\Gamma\left( \frac{1}{2}+\frac{1+\theta}{4} \right)}$$

$$=A\frac{\Gamma\left( \frac{1}{2} \right)\Gamma\left( 1 \right)}{\Gamma\left( \frac{3-\theta}{4} \right)\Gamma\left( \frac{3+\theta}{4} \right)}=\frac{A\sqrt{\pi}}{\Gamma\left( \frac{3-\theta}{4} \right)\Gamma\left( \frac{3+\theta}{4} \right)}=1.$$

We therefore have

$$g\left( z \right)=\frac{\Gamma\left( \frac{3-\theta}{4} \right)\Gamma\left( \frac{3+\theta}{4} \right)}{\sqrt{\pi}}F\left( -\frac{1-\theta}{4},-\frac{1+\theta}{4}; \frac{1}{2}; z \right), (A6)$$

or

$$\phi\left( x \right)=\frac{\Gamma\left( \frac{3-\theta}{4} \right)\Gamma\left( \frac{3+\theta}{4} \right)}{\sqrt{\pi}}F\left( -\frac{1-\theta}{4},-\frac{1+\theta}{4}; \frac{1}{2};\left( 2x-1 \right)^{2} \right). (A7)$$

Note that, for a complex number z, $\bar{\Gamma\left( z \right)}=\Gamma\left( \bar{z} \right)$. Therefore, if $\theta=\sqrt{1-8\mu}=i\omega$ is complex for $\mu>1/8$, $\Gamma\left( \frac{3+\theta}{4} \right)\Gamma\left( \frac{3-\theta}{4} \right)=\Gamma\left( \frac{3+\theta}{4} \right)\Gamma\left( \frac{3+\theta}{4} \right)\mathfrak{\in R}$.

The coefficient $a_{n}$ ($n>1$) of the hypergeometric function $F\left( -\frac{1-\theta}{4},-\frac{1+\theta}{4}; \frac{1}{2}; z \right)=1+\sum_{n=1}^{\infty} a_{n}z^{n}/n!$ is

$$a_{n}=\frac{\left( -\frac{1-\theta}{4} \right)_{n}\left( -\frac{1+\theta}{4} \right)_{n}}{\left( \frac{1}{2} \right)_{n}}.$$

The first few coefficients are

$$a_{1}=\frac{\left( -\frac{1-\theta}{4} \right)\left( -\frac{1+\theta}{4} \right)}{\frac{1}{2}}=\frac{1-\theta^{2}}{8}=\mu,$$

$$a_{2}=\frac{\left( -\frac{1-\theta}{4}+1 \right)\left( -\frac{1+\theta}{4}+1 \right)}{\frac{1}{2}+1}a_{1}=\frac{9-\theta^{2}}{3\cdot8}a_{1}=\frac{\left( 1+\mu\right)\mu}{3},$$

$$a_{3}=\frac{\left( -\frac{1-\theta}{4}+2 \right)\left( -\frac{1+\theta}{4}+2 \right)}{\frac{1}{2}+2}a_{2}=\frac{49-\theta^{2}}{5\cdot8}a_{2}=\frac{6+\mu}{5}a_{2}=\frac{\left( 6+\mu\right)\left( 1+\mu\right)\mu}{5\cdot3}.$$

In general

$$a_{n}=\frac{\left( -\frac{1-\theta}{4}+n-1 \right)\left( -\frac{1+\theta}{4}+n-1 \right)}{\frac{1}{2}+n-1}a_{n-1}=\frac{\left( 4n-5 \right)^{2}-\theta^{2}}{\left( 2n-1 \right)\cdot8}a_{n-1}$$

$$=\frac{\left( 4n-5 \right)^{2}-1+8\mu}{\left( 2n-1 \right)\cdot8}a_{n-1}=\frac{\left( 4n-4 \right)\left( 4n-6 \right)+8\mu}{\left( 2n-1 \right)\cdot8}a_{n-1}$$

$$=\frac{\left( n-1 \right)\left( 2n-3 \right)+\mu}{2n-1}a_{n-1}$$

Therefore

$$\phi\left( x \right)=A\left[ 1+\mu\left( 2x-1 \right)^{2}+\frac{\left( 1+\mu\right)\mu}{3}\frac{\left( 2x-1 \right)^{4}}{2!}+\cdots\right]$$

$$=A\left[ 1+\sum_{n=1}^{\infty} \frac{\prod_{j=1}^{n} \left( \left( j-1 \right)\left( 2j-3 \right)+\mu\right)}{\left( 2n-1 \right)‼}\frac{\left( 2x-1 \right)^{2n}}{n!} \right] (A8)$$

with

$$A=\frac{\Gamma\left( \frac{3-\sqrt{1-8\mu}}{4} \right)\Gamma\left( \frac{3+\sqrt{1-8\mu}}{4} \right)}{\sqrt{\pi}}. (A9)$$

The fixation probability $\phi\left( x \right)$ is a convex function of x, having the minimum

$\phi\left( \frac{1}{2} \right)=\frac{\Gamma\left( \frac{3-\sqrt{\theta}}{4} \right)\Gamma\left( \frac{3+\sqrt{\theta}}{4} \right)}{\sqrt{\pi}}$

at $x=1/2$, and the maximum 1 at $x=0$ and $x=1$.

In terms of the original parameter $\mu$, the minimum fixation probability at $x=1/2$ is

$\phi\left( \frac{1}{2} \right)=\frac{\Gamma\left( \frac{3-\sqrt{1-8\mu}}{4} \right)\Gamma\left( \frac{3+\sqrt{1-8\mu}}{4} \right)}{\sqrt{\pi}}$.

Note that

$\Gamma\left( \frac{3-\sqrt{1-8\mu}}{4} \right)=\Gamma\left( \frac{1}{2}+\mu+O\left( \mu^{2} \right) \right)=\sqrt{\pi}+\Gamma'\left( \frac{1}{2} \right)\mu+O\left( \mu^{2} \right)$

$=\sqrt{\pi}\left( 1+\psi\left( \frac{1}{2} \right)\mu+O\left( \mu^{2} \right) \right)$

and

$\Gamma\left( \frac{3+\sqrt{1-8\mu}}{4} \right)=\Gamma\left( 1-\mu+O\left( \mu^{2} \right) \right)=\sqrt{\pi}-\Gamma'\left( 1 \right)\mu+O\left( \mu^{2} \right)$

$=1-\psi\left( 1 \right)\mu+O\left( \mu^{2} \right),$

where

$$\psi\left( z \right)=\frac{\Gamma'\left( z \right)}{\Gamma\left( z \right)}$$

is a digamma function,

$$\psi\left( 1 \right)=-\gamma_{E}$$

$\psi\left( \frac{1}{2} \right)=-\gamma_{E}-2\ln2$,

and $\gamma_{E}=0.57721\cdots$ refers to the Euler constant (6.3.2 and 6.3.3 of Abramowitz and Stegun [1]). We therefore have

$\phi\left( \frac{1}{2} \right)=\frac{\sqrt{\pi}\left( 1+\psi\left( \frac{1}{2} \right)\mu-\psi\left( 1 \right)\mu+O\left( \mu^{2} \right) \right)}{\sqrt{\pi}}$,

$$=1-\left( 2\ln2 \right)\mu+O\left( \mu^{2} \right),$$

or

$P_{\mathrm{Emax}}=1-\phi\left( \frac{1}{2} \right)$

$$=\left( 2\ln2 \right)\mu+O\left( \mu^{2} \right)$$

$$=1.38629\times\mu+O\left( \mu^{2} \right). (A10)$$

## Reference

1. Abramowitz M, Stegun IA. Handbook of Mathematical Functions. New York: Dover Publications, Inc.; 1970.

## Appendix B: Branching process without frequency dynamics

We consider the branching process in which the number of reproduced infected cells by one infected cell is randomly determined with geometric distribution. Let $z_{t}$ be the probability that the double mutant has not been emerged until *t*-th generation starting with one 0-th generation infected cell. Then, we have $z_{0}=1$ and

$$z_{t}=\sum_{k=0}^{\infty} \frac{1}{1+m}\left( \frac{m}{1+m} \right)^{k}\left( \left( 1-u \right)^{n}z_{t-1} \right)^{k}$$

$$=\frac{1}{1+m-m\left( 1-u \right)^{n}z_{t-1}}, (t=1, 2, 3, \ldots)$$

where $m$ is the mean of geometric distribution. If we define $s_{t}$ as the probability that the double resistance emerges at *t*-th generation for the first time, it is calculated as follows

$$s_{t}=z_{t-1}-z_{t}. (t=1, 2, \ldots)$$

The probability of emergence for the whole process, $s$ is

$$s=\sum_{t=1}^{\infty} s_{t}$$

$$=z_{0}-z_{\infty}=1-z_{\infty},$$

and $z_{\infty}$ would satisfy

$$z_{\infty}=\frac{1}{1+m-m\left( 1-u \right)^{n}z_{\infty}}$$

$$m\left( 1-u \right)^{n}z_{\infty}^{2}-\left( 1+m \right)z_{\infty}+1=0$$

$$z_{\infty}=\frac{1+m\pm\sqrt{\left( 1+m \right)^{2}-4m\left( 1-u \right)^{n}}}{2m\left( 1-u \right)^{n}}$$

Since $0<z_{\infty}<1$,

$$z_{\infty}=\frac{1+m-\sqrt{\left( 1+m \right)^{2}-4m\left( 1-u \right)^{n}}}{2m\left( 1-u \right)^{n}},$$

which depends on $\left( 1-u \right)^{n}$. For sufficiently small $u$ and a finite $n$, $\left( 1-u \right)^{n}=1-nu+O(u^{2})$. Then, $z_{\infty}$ is approximately determined by $nu$. The values of $1-z_{\infty}$ are very close to the probability of emergence for the full model in the region of $nu$-scaling (upper right of Figure S2), which suggests the frequency dynamics of single-resistant genomes over successive infections is less effective on the emergence probability of double resistance in this region.

# Supplementary Figures

**Supplementary Figure 1. Comparison of emergence probability for branching process with that of diffusion approximation in WF model.**

The results of diffusion approximation (black line which has the slope of $2\ln2$) is added to Fig 6c. As $u$ becomes small, the results of diffusion approximation converges to other results.

**Supplementary Figure 2.** **Emergence probability for full model is compared with that without frequency dynamics.**

For both figures, the mean number of new infections from an infected cell, $m$ is 1. (**a**) Contour plot of the emergence probability, which is the same as Fig. 7a. (**b**) Contour plot of $1-z_{\infty}$ calculated in Appendix B in Supplementary material.

**Supplementary Figure 3. Contour plots of emergence probability when** $\boldsymbol{m}$ **is close to 1.**

For each panel, the mean number of new infections from an infected cell, $m$ is (a) 0.9, (b) 0.95, (c) 0.99, (d) 1.01, (e) 1.05, (f) 1.1, respectively. In call cases, the initial frequency of single mutant is set to be 0.5.

**Supplementary Figure 4. The slopes of the contours in the** $\boldsymbol{n}$**-**$\boldsymbol{u}$ **plane.**

The parameters of each panel corresponds to the panel shown in Supplementary Figure 3. The points with slope of lower than –2 are shown with black.
